# Adolescent mental health services in West Africa: a comparative analysis of Burkina Faso, Ghana, and Niger

**DOI:** 10.1186/s13034-024-00827-8

**Published:** 2024-10-14

**Authors:** Michel Adurayi Amenah, Nassirou Ibrahim, Ludovic Deo Gracias Tapsoba, Jacob Novignon, Ama Pokuaa Fenny, Irene A. Agyepong, Roxane Borges da Silva, Tim Ensor

**Affiliations:** 1 Ghana College of Physicians and Surgeons, Accra, Ghana; 2 Laboratory for Studies and Research on Social Dynamics and Local Development, Ouagadougou, Burkina Faso; 3 University of Montreal, Montreal, QC Canada; 4 Kwame Nkrumah University of Science and Technology, Kumasi, Ghana; 5 Institute of Statistical Social and Economic Research, University of Ghana, Accra, Ghana; 6 Dodowa Heath Research Centre, Dodowa, Ghana; 7 University of Leeds, Leeds, UK

**Keywords:** Adolescents, Mental health, West Africa

## Abstract

**Background:**

Adolescent mental health (AMH) is a critical issue worldwide, particularly in West Africa, where it is intensified by socio-economic, cultural, and security challenges. Insecurity and the presence of mining sites expose adolescents to hazardous environments, substance abuse, and adulterated alcohol, further aggravating their mental health. Despite these severe issues, research on AMH in this region remains limited. This study aims to analyze the provision of AMH services in Burkina Faso, Ghana, and Niger, highlighting the unique challenges these countries face within the broader West African healthcare context.

**Methods:**

The study adopted a multi-stage, stratified sampling design to collect data from primary healthcare centers (PHCs) in the three countries. Using STATA.17, Descriptive analysis was conducted on the data related to availability of AMH services, types of mental health disorders treated, resources available, and OPD attendance rates. The analysis also incorporated factors such as the rural-urban divide and the presence of national guidelines for AMH services.

**Results:**

The findings reveal a significant shortfall in the provision of AMH services across the region, with less than 30% of PHCs across all the countries offering these services. The study also highlights a pronounced rural-urban disparity in AMH service availability, a general absence of national guidelines for AMH care, and low OPD attendance rates.

**Conclusion:**

The study highlights the urgent need for comprehensive policy reform and targeted interventions to enhance AMH services in West Africa. Key policy reforms should include the development and implementation of national guidelines for AMH care and integration of AMH services into primary healthcare. Additionally, efforts should focus on capacity building through the training of mental health professionals, increasing public awareness to reduce stigma, and ensuring equitable resource allocation across rural and urban areas. Improving AMH care is essential not only for the well-being of adolescents but also for driving broader socio-economic development in the region.

## Background

Globally, adolescents represent a vital demographic, accounting for 16% of the world’s population, with 1.3 billion people [1]. In West and Central Africa, this demographic is particularly significant, representing about 23% of the population, with projections indicating an increase to 32% by 2030 [2]. In Ghana, adolescents aged 10 to 19 years comprise roughly 22% of the national populace [3]. In Niger, adolescents form over 24% of the population [4], while in Burkina Faso, youth under 25 represent two-thirds of the populace, highlighting the region’s youthful demographic and the importance of targeted healthcare and social services [5].

Adolescence, a pivotal life stage from 10 to 19 years, is marked by substantial physiological, psychological, physical, and social transformations, including sexual maturation. This phase is instrumental in shaping habits concerning diet, physical activity, substance use, and sexual behavior, which can profoundly influence future health outcomes [6]. Mental health emerges as a significant issue during this period. The World Health Organization (WHO) underscores the severe consequences of mental health problems on adolescents, noting that 1.1 million adolescents die each year from causes such as suicide and interpersonal problems [7].

According to the WHO, mental health disorders such as unipolar depressive disorders and anxiety disorders are among the leading causes of illness and disability among adolescents [8]. Suicide, which is often linked to mental health problems, is the fourth leading cause of death among 15–29-year-olds [8]. Additionally, alcohol use disorders are among the top causes of years lost to disability for males aged 15–19 years [9]. Early onset of substance use is associated with higher risks of developing dependence and other problems during adult life [8].

Mental health concerns among adolescents in sub-Saharan Africa are alarmingly prevalent, as evidenced by a systematic review covering 97,616 adolescents [10]. This review reveals high median point prevalence for various mental health issues including depression, anxiety disorders, emotional and behavioral problems, Post-Traumatic Stress Disorder (PTSD), and suicidal ideation, underscoring the critical need for intervention in this demographic [11]. In addition, another systematic review by Cortina et al. [10] found that between 12% and 30% of children and young people aged 5 to 24 years had mental health problems.

The escalating concern for Adolescent Mental Health (AMH) in West Africa stems from a blend of unique socio-economic, cultural, and healthcare challenges [12, 13]. In countries like Burkina Faso, Ghana, and Niger, these factors intertwine to create a mental health landscape that’s distinct from global narratives. While global mental health discourses have advanced, these West African nations still confront significant hurdles. Socio-economic hardships exacerbate mental health issues among adolescents, who often navigate these challenges amidst deeply ingrained cultural beliefs and stigmas surrounding mental health [13–15]. This backdrop is compounded by limited healthcare resources, which impede the availability and accessibility of adequate mental health services. The resultant impact on adolescents is profound, influencing not only their immediate mental well-being but also their long-term health and socio-economic prospects.

The significance of mental health disorders in adolescents, especially during a critical developmental stage, cannot be overstated. However, in West Africa, the scenario is more challenging due to the under-prioritization of AMH services within healthcare systems strained by limited resources [16]. This region, characterized by high poverty, political instability, and deep-rooted cultural barriers, faces added complexities in addressing these mental health issues [13]. The intersection of socio-economic hardships with cultural perceptions often leads to inadequate recognition and management of mental health disorders, further impeding the development of effective mental health support systems for adolescents. This intricate situation demands a nuanced approach to understanding and tackling adolescent mental healthcare in West Africa.

The diverse socio-cultural landscapes and healthcare provisions of Burkina Faso, Ghana, and Niger make them compelling cases for studying AMH. Ghana’s progress in mental health legislation, particularly with the 2012 Mental Health Act, is a significant step towards service improvement and stigma reduction [16, 17]. However, challenges persist in the uniform implementation of these policies across different regions.

In Burkina Faso, the mental health landscape is shaped by a combination of ongoing conflict, displacement, and poverty, which have led to a high prevalence of psychological suffering among the population [18]. The government passed a mental health policy in 2020, but as of 2021, it had not yet provided any human or financial resources for its implementation [18]. The country has a significant lack of mental health professionals, with only 103 mental health professionals for the entire population [18]. The Mental Health Atlas observed a 112-time increase in the number of community-based mental health services per 100,000 people between 2014 and 2020, suggesting that many citizens are seeking help [18].

In Niger, the situation is similar, with several barriers to improving mental health services, including long distances to the nearest hospital and a lack of funding for home-based visits by health workers [19] This study was the first step in Niger’s plan to implement the WHO’s Mental Health Gap Action Programme (mhGAP) at a national level [19]. The cultural context in Niger and Burkina Faso significantly influences mental health outcomes, as traditional beliefs about mental illness, often rooted in the idea that mental disorders are caused by supernatural forces or transgressions of societal taboos, complicate the process of seeking care [20, 21]. Stigmatization of mental illness, coupled with these traditional beliefs, discourages individuals from seeking help or receiving appropriate care. The reliance on traditional healers and religious figures, who are often seen as the primary sources of intervention due to the scarcity of formal mental health services, further hinders access to evidence-based care [22]. This situation is exacerbated by the limited awareness and understanding of mental health issues among the general population, including policymakers, which impedes the development of effective mental health policies and programs [23–25].

Furthermore, political instability and economic constraints in these countries contribute to the overall challenge of addressing AMH needs [18]. Mental health often takes a backseat in national health agendas focused on more immediate public health crises, such as infectious diseases and malnutrition [26]. This leads to a cycle where mental health remains marginalized, both in terms of policy attention and resource allocation.

The study’s primary objective is to examine and compare the state of AMH services in these three countries. Key research questions are: (1) How do the existing mental health services cater to the needs of adolescents in Burkina Faso, Ghana, and Niger? (2) What are the primary barriers to accessing mental healthcare for adolescents in these countries?

By addressing these questions, this research aims to shed light on the critical gaps and opportunities in AMH services in West Africa. The study’s findings are expected to provide valuable insights for policymakers, healthcare providers, and mental health advocates to improve the mental health outcomes for adolescents in this region.

## Methods

### Data

The data utilized in this study was sourced from a comprehensive field survey conducted as part of the Adolescent Mental, Sexual and Reproductive Health and Wellbeing Policy, Program and Primary Care Implementation Priorities in West Africa (AdoWA) project. The focus of this project was on assessing the implementation priorities for policies, programs, and primary care in the domains of mental, sexual, and reproductive health, as well as the overall well-being of adolescents in Burkina Faso, Ghana, and Niger. A significant aspect of this project was its quantitative analysis of health structures in each of these countries. For this quantitative component, data collection was carried out through structured surveys of PHC facilities in each country, aiming to gather robust and relevant data pertinent to the project’s objectives.

### Sampling

The study employed a systematic, multi-stage sampling design to ensure an analysis reflective of diverse socio-economic and geographical settings. This methodology was standardized across Ghana, Burkina Faso, and Niger, with adjustments tailored to each country’s specific context. The design encompassed region, district, and facility selection stages to capture a broad spectrum of health service provision landscapes. In Ghana, the Greater Accra Region was selected for its urban-rural mix, with four districts chosen to highlight service disparities. Burkina Faso’s study focused on the West-Central and Hauts Bassins regions, selecting five urban and two rural districts for their accessibility and health indicators. Niger’s approach targeted Maradi and Niamey, aiming for a balanced urban-rural representation. This uniform sampling framework across countries, designed to ensure geographic and demographic representation, was carefully considered to balance scientific rigor with practical constraints such as accessibility and budget.

### Ghana

In conducting the study in Ghana, a systematic, multi-stage sampling design was employed to accurately represent the diverse landscape of adolescent mental, sexual, and reproductive health services. This process comprised three distinct stages: region selection, district selection, and facility selection.

In the first stage, the Greater Accra Region was purposively chosen as the primary study area, owing to its demographic diversity and the presence of both urban and rural districts. This region mirrors the broader socio-economic, healthcare, and population dynamics of Ghana, making it an ideal location for a comprehensive analysis of adolescent health services across various settings. The inclusion of Greater Accra thus provides valuable insights into the national context of adolescent healthcare.

Consequently, four districts within the Greater Accra Region were randomly selected to illustrate the disparities in mental health service provision between urban and rural areas. This selection was based on a set of criteria, including demographic characteristics, healthcare infrastructure, and existing disparities in adolescent health services. The chosen districts—Ga East and La Kwantemang representing urban areas, and Ningo Prampram and Shai Osudoku representing rural areas—highlight the socio-economic and healthcare challenges unique to each setting. This step is crucial in understanding how geographical location and the degree of urbanization influence the availability and quality of AMH services.

The final stage involved the inclusion of all functional PHC facilities and senior high school sick bays within the selected districts, accounting for a total of 80 PHC facilities. This approach was adopted to ensure that the study encapsulates a broad spectrum of service provision models, sizes of PHC facilities, and resource allocations. By covering a wide range of healthcare settings, the study aims to provide a holistic view of the adolescent health service landscape in these districts.

### Burkina Faso

For the study conducted in Burkina Faso, a three-stage sampling process was employed. In the initial stage, two regions - Centre-Quest and Hauts Bassins - were purposively chosen. The selection criteria for these regions were twofold: the ease of accessibility in Burkina Faso insecurity context and specific health indicators, particularly those relating to mental, sexual, and reproductive health.

Proceeding to the second stage, three urban and two rural districts were selected systematically at random. For the Centre-Quest, the urban districts were Léo, Réo and Koudougou and the rural districts were Tenado and Sabou. For the Hauts Bassins region, the urban districts were Dafra, Orodara, Hounde and the rural districts are Dandé and Karangasso Vigue.

In the final stage of the sampling process, a total of 160 PHC facilities were selected, 80 PHC facilities in each region. This selection encompassed a diverse range of healthcare providers, ensuring a broad representation of the health services landscape in these regions of Burkina Faso.

### Niger

In the Niger segment of the study, a structured stratified, multistage sampling design was employed, focusing on two key regions: Maradi and Niamey. These regions were purposively selected due to their contrasting characteristics concerning adolescent mental, sexual, and reproductive health indicators, as well as the distribution of urban and rural PHC facilities.

Within the selected regions of Maradi and Niamey, five health districts each were chosen to ensure a balanced representation of both urban and rural healthcare environments. This phase of the sampling process was executed using a blend of purposive and random sampling methods. In Maradi, districts were carefully selected to capture the diversity and challenges of rural healthcare. In Niamey, the focus was on understanding the urban healthcare dynamic. This selection process was pivotal in ensuring a representative and diverse sample that could accurately reflect the healthcare realities in different parts of Niger.

The study included a total of 160 PHC facilities across the two regions, ensuring an equitable distribution between Maradi and Niamey. The process for selecting these PHC facilities was twofold: firstly, a quota system was implemented for private PHC facilities, and secondly, a proportional random selection method was used for public PHC facilities. This approach was designed to ensure a comprehensive and representative mix of the various types of healthcare infrastructure present in both urban and rural settings of the selected regions.

### Data analysis

The first phase of the analysis focused on descriptive statistics to establish a foundational understanding of the data. This involved calculating central tendency measures (means, medians) and dispersion measures (standard deviations, ranges) for quantitative variables. For categorical variables, frequency distributions were computed. This step was essential to identify basic trends, patterns, and characteristics within the dataset, providing a preliminary snapshot of the mental, sexual, and reproductive health services landscape in the surveyed regions.

Building on the descriptive analysis, the study then transitioned into a comparative analysis, given its focus on three distinct countries: Burkina Faso, Ghana, and Niger. This stage was critical in highlighting the similarities and differences in health service provision across these nations. Comparative analysis involved examining key indicators such as the availability and accessibility of health services, and the quality of healthcare PHC facilities in each country. By juxtaposing these indicators across Burkina Faso, Ghana, and Niger, the study aimed to uncover unique regional characteristics and shared challenges in adolescent health service provision. Descriptive statistics were computed using Stata version 17.0 (Stata MP/StataCorp LLC) [27].

## Results

### Demographic statistics

Table 1 delineates the distribution of PHC facilities by location and type across Ghana, Niger, and Burkina Faso, alongside the gender demographics of facility leadership and the nature of operating authorities.

The rural-urban divide shows a higher inclination towards rural PHC facilities within the collective dataset, with 57.91% of PHC facilities located in rural areas compared to 42.09% in urban settings. Ghana notably diverges from this trend, with a majority (62.50%) of its sampled PHC facilities situated in urban areas. The majority of the PHC facilities visited in Niger and Burkina Faso were located in rural areas, with 55.00% in Niger and 71.71% in Burkina Faso.

Health Centers serve as the backbone of health service provision, accounting for 81.54% of all PHC facilities across the samples for the three countries. This dominance is particularly pronounced in Niger and Burkina Faso, where Health Centers account for 96.88% and 94.73% of PHC facilities, respectively. Ghana’s sample, however, shows a more diverse health service structure, with Community-based Health Planning and Services (CHPS) compounds (26.25%) and Clinics (37.50%) complementing the traditional Health Center model.

82.86% of the PHC facilities surveyed across the countries. In Niger and Burkina Faso, over 90% of the PHC facilities are under government operation, indicating a strong state-led health service framework. In contrast, Ghana, particularly in the Greater Accra region, presents a more balanced public-private dynamic, with private PHC facilities constituting half of the health service providers in this sample. However, this balance may not reflect the situation in other regions of Ghana, where government-operated PHC facilities might play a more dominant role.

**Table 1:** Demographic distribution of PHC facilities across countries

Variables	All Countries		Ghana		Niger		Burkina Faso	
	Freq.	Percent	Freq.	Percent	Freq.	Percent	Freq.	Percent
*Location*								
Urban	165	42.09	50	62.50	72	45.00	43	28.29
Rural	227	57.91	30	37.50	88	55.00	109	71.71
*Type of facility*								
Health center	319	81.34	20	25.00	155	96.88	144	94.73
CHPS	28	7.18	21	26.25	-	-	-	-
Clinic	36	9.28	30	37.50	5	3.13	8	5.27
Sick bays	9	2.20	9	11.25		-	-	
*Operating authority*								
Government	325	82.28	38	47.50	148	92.50	139	91.45
Mission	10	2.53	2	2.50	2	1.25	6	3.95
Private	53	13.42	40	50.00	6	3.75	7	4.61
Other (Specify)	4	1.01	-	-	4	2.50	-	-
*Gender (Head)*								
Male	237	60.46	28	35.00	96	60.00	113	74.34
Female	155	39.54	52	65.00	64	40.00	39	25.66

Ghana: Greater Accra (Ga East, La Kwantemang, Ningo Prampram, Shai Osudoku), Niger: Niamey (District sanitaire de Niamey I, District sanitaire de Niamey II, District sanitaire de Niamey III, District sanitaire de Niamey IV, District sanitaire de Niamey V), Maradi (District sanitaire de Dakoro, District sanitaire de Madarounfa, District sanitaire de Maradi ville, District sanitaire de Mayahi, District sanitaire de Tessaoua), Burkina Faso: Centre-Quest (Léo, Réo, Koudougou, Tenado, Sabou), Hauts Bassins (Dafra, Orodara, Hounde, Dandé, Karangasso Vigue)

Gender representation among the heads of PHC facilities is skewed towards males overall (60.46%). However, Ghana contrasts sharply with its counterparts, with a majority of female-headed PHC facilities (65.00%). This could be due to Ghana’s proactive gender equality policies and targeted initiatives that encourage and support women in leadership roles, particularly in the healthcare sector [28, 29]. Niger and Burkina Faso follow the general trend with a male-dominant leadership in their PHC facilities, with females heading 40% and 25.66% of the PHC facilities, respectively.

### Adolescent mental health services

Table 2 details the availability and capacity of PHC facilities across the selected districts of Ghana, Niger, and Burkina Faso to provide AMH services and address specific mental disorders. The data is pivotal in understanding the regional disparities and healthcare capabilities within these West African countries.

Out of a total of 392 surveyed PHC facilities, only 103 (26.28%) offer AMH services. The country-specific breakdown reveals notable differences: in the selected districts from Ghana, 23 out of 80 PHC facilities (28.75%) provide these services, while selected districts in Niger, with 15 out of 160 PHC facilities (9.38%), shows the lowest availability. The districts from Burkina Faso lead in service provision, with 65 out of 152 PHC facilities (42.76%) offering AMH services. This stark contrast among the countries highlights the varied emphasis and resource allocation towards adolescent mental healthcare.

**Table 2:** AMH Services in PHC facilities across countries

Variables	All Countries		Ghana		Niger		Burkina Faso	
	Freq.	Percent	Freq.	Percent	Freq.	Percent	Freq.	Percent
*Offer AMH services*								
Yes	103	26.28	23	28.75	15	9.38	65	42.76
No	289	73.72	57	71.25	145	90.63	87	57.24
*Basic counselling/psychosocial support services*								
Yes	95	92.23	22	95.65	10	66.67	63	96.92
No	8	7.77	1	4.35	5	33.33	2	3.08
*Laboratory*								
Yes	24	23.30	13	56.52	0	0	11	16.92
No	79	76.70	10	43.48	15	100.00	54	83.08
*First line oral psychoactive medications*								
Yes	36	34.95	12	52.17	6	40.00	18	27.69
No	67	65.05	11	47.83	9	60.00	47	72.31
*Emergency services (acute psychosis, suicide attempt)*								
Yes	48	46.60	12	52.17	4	26.67	32	49.23
No	55	53.40	11	47.83	11	73.33	33	50.77

Ghana: Greater Accra (Ga East, La Kwantemang, Ningo Prampram, Shai Osudoku), Niger: Niamey (District sanitaire de Niamey I, District sanitaire de Niamey II, District sanitaire de Niamey III, District sanitaire de Niamey IV, District sanitaire de Niamey V), Maradi (District sanitaire de Dakoro, District sanitaire de Madarounfa, District sanitaire de Maradi ville, District sanitaire de Mayahi, District sanitaire de Tessaoua), Burkina Faso: Centre-Quest (Léo, Réo, Koudougou, Tenado, Sabou), Hauts Bassins (Dafra, Orodara, Hounde, Dandé, Karangasso Vigue)

A high percentage of PHC facilities (92.23%) offered basic counselling and psychosocial support services across the selected districts. Selected districts from Ghana lead with 22 out of 23 PHC facilities (95.65%) providing these services, followed closely by selected districts from Burkina Faso with 63 out of 65 PHC facilities (96.92%), while selected districts from Niger trail with 10 out of 15 PHC facilities (66.67%). This indicates a strong regional emphasis on counselling and psychosocial support for adolescents.

Only 24 PHC facilities (23.30%) across all selected districts from all countries provide laboratory screening for substance use. Selected districts from Ghana demonstrate a higher capacity, with 13 PHC facilities (56.52%) offering these services, whereas selected districts from Niger have no PHC facilities providing such screenings. Selected districts from Burkina Faso have 11 out of 65 PHC facilities (16.92%) equipped for this purpose, highlighting a critical shortfall in laboratory support.

Across the selected districts from all countries, 36 PHC facilities (34.95%) offer first-line oral psychoactive medications like Prozac. Selected districts from Ghana lead with 12 PHC facilities (52.17%), followed by selected districts from Niger with 6 PHC facilities (40.00%), and selected districts from Burkina Faso with 18 PHC facilities (27.69%), showcasing varying degrees of medication availability in the region.

Emergency services for conditions like acute psychosis and suicide attempts are available in 48 PHC facilities (46.60%) across the selected districts from all countries. Selected districts from Ghana have the highest provision rate with 12 PHC facilities (52.17%), followed by selected districts from Burkina Faso with 32 PHC facilities (49.23%), and selected districts from Niger with the lowest provision at 4 PHC facilities (26.67%).

### Skills and resources in provision of AMH services

Table 3 provides an overview of the availability of essential skills and resources for AMH services in PHC facilities across the selected districts from Ghana, Niger, and Burkina Faso. The study focuses on the presence of national guidelines for mental health services, staff training in AMH services, the provision of basic counseling and psychosocial support, laboratory screening for substance use, availability of first-line oral psychoactive medications like Prozac, and emergency services for acute conditions such as psychosis and suicide attempts.

This metric evaluates whether PHC facilities possess national guidelines for mental health service provision to adolescents. Only 22 out of 103 PHC facilities offering AMS (21.36%) across the selected districts have these guidelines. In the selected districts from Ghana, this is observed in 4 out of 23 PHC facilities (17.39%), Niger shows a higher availability with 7 out of 15 PHC facilities (46.67%), and Burkina Faso has these guidelines in 11 out of 65 PHC facilities (16.92%). Most PHC facilities across all selected districts, therefore, do not have these national guidelines (78.64%).

The study aimed to assess the capacity and skills of healthcare professionals within various PHC facilities to manage a range of psychiatric conditions, specifically targeting alcohol and substance use disorders, depression, anxiety disorders, suicidality and self-harm, and psychotic disorders. When it comes to treating Alcohol and Substance Use Disorders, 58 out of the 103 PHC facilities across all selected districts (56.31%) are equipped to handle these conditions. In the selected districts from Ghana, a significant majority (69.57%) of the AMH-providing PHC facilities can treat these disorders. Niger reports a similar high capacity (73.33%), while Burkina Faso lags slightly behind at 47.69%. These figures indicate a relatively strong focus on addressing substance-related issues in the region’s adolescent population.

Also, for depression treatment, 71 PHC facilities (68.93%) across the selected districts from all countries have staff skilled in providing such services. Selected districts from Ghana again show a higher skilled capacity, with 20 out of 23 PHC facilities (86.96%) equipped to handle depression. Niger follows with 12 out of 15 PHC facilities (80.00%), and selected districts from Burkina Faso have 39 out of 65 PHC facilities (60.00%) providing care for depression. The high percentages across the board suggest a regional acknowledgment of depression as a critical aspect of AMH.

**Table 3:** Skills and resources in the provision of AMS in PHC facilities across countries

Variables	All Countries		Ghana		Niger		Burkina Faso	
	Freq.	Percent	Freq.	Percent	Freq.	Percent	Freq.	Percent
*National guidelines for mental health services*								
Yes	22	21.36	4	17.39	7	46.67	11	16.92
No	81	78.64	19	82.61	8	53.33	54	83.08
*Alcohol and substance use*								
Yes	58	56.31	16	69.57	11	73.33	31	47.69
No	45	43.69	7	30.43	4	26.67	34	52.31
*Depression*								
Yes	71	68.93	20	86.96	12	80.00	39	60.00
No	32	31.07	3	13.04	3	20.00	26	40.00
*Anxiety disorders*								
Yes	67	65.05	19	82.61	8	53.33	40	61.54
No	36	34.95	4	17.39	7	46.67	25	38.46
*Suicidality and self-harm*								
Yes	49	47.57	13	56.52	3	20.00	33	50.77
No	54	52.43	10	43.48	12	80.00	32	49.23
*Psychotic disorders*								
Yes	52	50.49	14	60.87	5	33.33	33	50.77
No	51	49.51	9	39.13	10	66.67	32	49.23

Ghana: Greater Accra (Ga East, La Kwantemang, Ningo Prampram, Shai Osudoku), Niger: Niamey (District sanitaire de Niamey I, District sanitaire de Niamey II, District sanitaire de Niamey III, District sanitaire de Niamey IV, District sanitaire de Niamey V), Maradi (District sanitaire de Dakoro, District sanitaire de Madarounfa, District sanitaire de Maradi ville, District sanitaire de Mayahi, District sanitaire de Tessaoua), Burkina Faso: Centre-Quest (Léo, Réo, Koudougou, Tenado, Sabou), Hauts Bassins (Dafra, Orodara, Hounde, Dandé, Karangasso Vigue)

The capacity to treat anxiety disorders is present in 67 PHC facilities (65.05%) across all selected districts. The selected districts from Ghana top the list with 19 out of 23 PHC facilities (82.61%) offering such treatment, while those from Niger and Burkina Faso show capacities of 53.33% and 61.54%, respectively. This demonstrates a substantial regional recognition of anxiety disorders as a significant concern in AMH.

Regarding Suicidality and Self-Harm, 49 PHC facilities (47.57%) across all selected districts provide necessary care. The selected districts from Ghana have 13 PHC facilities (56.52%) addressing these issues, compared to only 3 PHC facilities in the selected districts from Niger (20%). The selected districts from Burkina Faso report care availability in 33 out of 65 PHC facilities (50.77%), indicating a moderate focus on these critical issues.

Lastly, treatment for Psychotic Disorders is available in 52 PHC facilities (50.49%) across the surveyed selected districts. The selected districts from Ghana have the highest capacity with 14 PHC facilities (60.87%), while those from Niger and Burkina Faso report capacities of 33.33% and 50.77%, respectively. This distribution suggests a balanced approach towards psychotic disorders in AMH across these regions.

### Rural vs. urban

Table 4 analysis provides a comparison of the availability and capacity for AMH services in urban and rural PHC facilities across the selected districts from Ghana, Burkina Faso, and Niger, encompassing a range of mental health conditions.

**Table 4:** Comparison of AMH Services in Urban and Rural PHC facilities across the countries

Variables	All Countries		Ghana		Niger		Burkina Faso	
	Urban	Rural	Urban	Rural	Urban	Rural	Urban	Rural
*Offer AMH services*								
Yes	46 (27%)	57 (25%)	13 (26%)	10(33%)	5 (7%)	10 (11%)	28 (65%)	37 (34%)
No	119 ( 73%)	170 (75%)	37 (74%)	20(66%)	67(93%)	78 (89%)	15 (35%)	72 (66%)
*Alcohol and substance use disorders*								
Yes	25 (54%)	33 (58%)	7 (54%)	9 (90%)	1 (20%)	10 (100%)	17 (61%)	14 (38%)
No	21 (46%)	24 (42%)	6 (46%)	1 (10%)	4 (82%)	0	11 (39%)	23 (62%)
*Depression*								
Yes	35 (76%)	36 (63%)	10 (77%)	10 (100%)	4 (80%)	8 (80%)	21 (75%)	18 (49%)
No	11 (24%)	21 (37%)	3 (23%)	0	1 (20%)	2 (20%)	7 (37%)	19 (51%)
*Anxiety disorders*								
Yes	35 (76%)	32 (56%)	11 (85%)	8 (80%)	4 (80%)	4 (40%)	20 (71%)	20 (54%)
No	11 (24%)	25 (44%)	2 (15%)	2 (20%)	1 (20%)	6 (60%)	8 (29%)	17 (46%)
*Suicidality and self-harm*								
Yes	23 (50%)	23 (43%)	5 (38%)	8 (80%)	1 (20%)	2 (20%)	17 (61%)	16 (43%)
No	23 (50%)	31 (57%)	8 (62%)	2 (20%)	4 (80%)	8 (80%)	11 (39%)	21 (57%)
*Psychotic disorders*								
Yes	21 (46%)	31 (54%)	5 (38%)	9 (90%)	1 (20%)	4 (40%)	15 (54%)	18 (49%)
No	25 (54%)	26 (46%)	8 (62%)	1 (10%)	4 (80%)	6 (60%)	13 (46%)	19 (51%)

Ghana: Greater Accra (Ga East, La Kwantemang, Ningo Prampram, Shai Osudoku), Niger: Niamey (District sanitaire de Niamey I, District sanitaire de Niamey II, District sanitaire de Niamey III, District sanitaire de Niamey IV, District sanitaire de Niamey V), Maradi (District sanitaire de Dakoro, District sanitaire de Madarounfa, District sanitaire de Maradi ville, District sanitaire de Mayahi, District sanitaire de Tessaoua), Burkina Faso: Centre-Quest (Léo, Réo, Koudougou, Tenado, Sabou), Hauts Bassins (Dafra, Orodara, Hounde, Dandé, Karangasso Vigue)

The findings reveal a complex landscape where AMH services show slightly higher availability in rural areas (57%) compared to urban settings (46%) across the selected districts in the surveyed countries. In Ghana, a marginally higher provision of AMH services is observed in urban areas (26%), contrasting with the rural-focused trends seen in Niger and Burkina Faso. The results indicates that urban provision of AMH services is lower in Niger (7%) compared to rural areas (11%), this might reflect a strategic focus on addressing healthcare gaps in rural regions, where access to services is typically more limited. This rural emphasis could be due to targeted interventions by the government or NGOs to improve healthcare availability in underserved areas [30]. Conversely, Burkina Faso’s substantial urban concentration of AMH services (65% in urban areas compared to 34% in rural settings) likely results from the concentration of resources, infrastructure, and trained professionals in urban centers, which are often prioritized for healthcare investments due to their population density and accessibility [31, 32].

When analyzing specific mental health conditions, the data shows a general rural inclination for treating alcohol and substance use disorders, except in Burkina Faso, where urban PHC facilities (61%) surpass rural ones (38%) in providing treatment. Depression treatment shows a relatively balanced approach between urban and rural PHC facilities, with a slight preference for rural settings in Ghana and Burkina Faso. Anxiety disorders are addressed with nearly equal emphasis in both settings, although Ghana has a slightly higher urban capacity. Notably, PHC facilities addressing suicidality, self-harm, and psychotic disorders are more prevalent in rural areas across all countries, underscoring a critical focus on rural mental health needs.

### Outpatient department (OPD) attendance for AMH services (2018–2021)

**Fig. 1:**
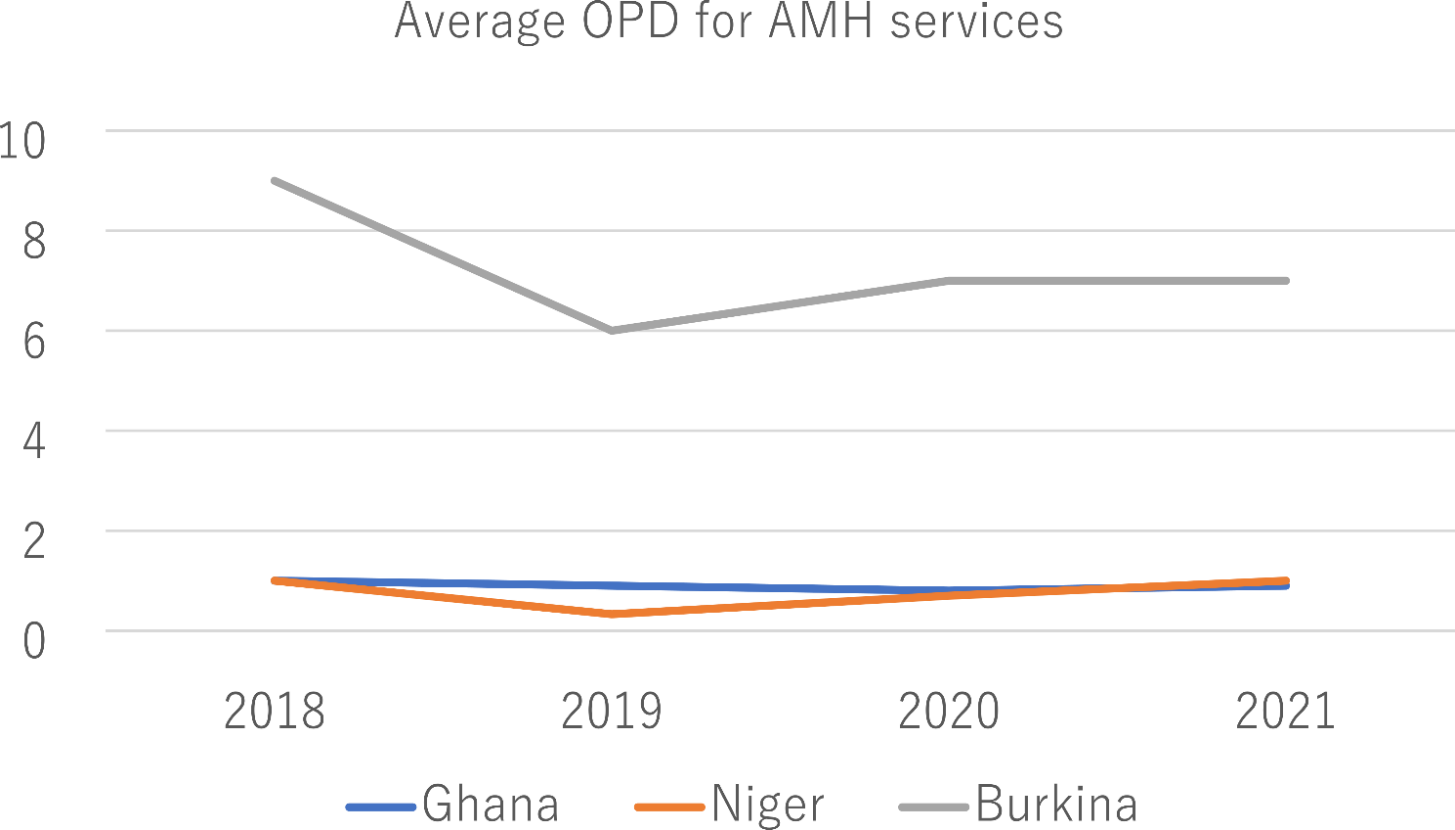
Trends in average outpatient visits for adolescent mental health services (2018–2021)

Figure 1 illustrates the trend of outpatient department (OPD) visits for adolescent mental health (AMH) services from 2018 to 2021 across three countries: Ghana, Niger, and Burkina Faso. In the selected districts of Ghana, among the 23 primary health care (PHC) centers providing AMH services, only 8 recorded OPD visits. In Niger, 15 of the surveyed PHC facilities reported OPD visits. Conversely, in Burkina Faso, a more significant number of 63 PHC facilities documented attendance for AMH services, reflecting a higher level of engagement in AMH care in that country.

### Source: authors’ computations

Between 2018 and 2021, the average number of OPD visits for AMH services in the selected districts of Ghana and Niger was notably low, with PHC facilities in both countries averaging one case or none at all during this period. In stark contrast, the selected districts in Burkina Faso began with a higher average of 9 OPD visits in 2018, which decreased to an average of 7 visits by 2021. This trend indicates a decline in OPD visits for AMH services in Burkina Faso over the four years, while Ghana and Niger remained consistently low throughout the same timeframe.

## Discussion

The analysis of adolescent AMH services in some selected districts from Ghana, Burkina Faso, and Niger reveals a pervasive shortfall across these West African countries. The overall low provision of AMH services across the selected districts from all the countries, as illustrated in Table 2, highlights a systemic gap within the healthcare infrastructure. With the highest rate observed in Burkina Faso at 42.76% and the lowest in Niger at a mere 9.38%, it is evident that the majority of PHCs are not equipped to meet the mental health needs of their adolescent populations. This lack of services is concerning, given the WHO’s identification of neuropsychiatric disorders as a leading cause of disability in young people [8]. The regional disparities suggest that while some efforts are being made, they are not widespread enough to create a robust safety net for adolescents in need of mental healthcare.

The availability of AMH services in West Africa is hindered by a complex interplay of socio-cultural and economic challenges. Cultural stigmas surrounding mental health often led to the marginalization of those suffering from mental disorders. In many West African societies, mental health issues are inaccurately attributed to supernatural causes or personal failings, leading to social ostracization, and discouraging individuals from seeking necessary care [15]. This situation is exacerbated by a lack of public awareness and education about mental health, further entrenching misconceptions and stigma.

Access to mental healthcare in the region is severely hindered by the acute scarcity of trained mental health professionals. The shortage of skilled personnel is driven by several factors, including limited training opportunities and the absence of specialized mental health education programs [33]. Moreover, the migration of trained professionals to regions with better opportunities exacerbates this deficit, leaving primary healthcare centers understaffed and ill-equipped to provide adequate mental health services [22, 34]. This lack of skilled personnel compromises the quality of care available to adolescents, further deterring individuals from seeking treatment. Additionally, cultural stigma surrounding mental health continues to be a significant barrier, preventing many from accessing the services they need [35]. In many communities, mental health issues are misunderstood or dismissed, leading to social ostracization and discouraging individuals from seeking help [36]. This cultural challenge is compounded by a general lack of awareness and education about mental health, which further entrenches misconceptions and limits the effectiveness of existing services.

The rural-urban divide in the provision of AMH services in West Africa, as highlighted in Table 1, underscores a significant challenge in resource allocation and healthcare equity. Urban centers, particularly in Ghana, demonstrate a relatively better availability of AMH services. This trend, however, starkly contrasts with the situation in rural areas of Burkina Faso and Niger, where a substantial number of PHC facilities are based but remain inadequately equipped to offer specialized mental healthcare.

This disparity is indicative of broader issues in healthcare planning and resource distribution. Urban areas, often with greater population density and visibility, tend to attract more investment and healthcare resources, including trained professionals and specialized services. In contrast, rural areas, despite housing a significant portion of the population, frequently suffer from neglect in healthcare resource allocation. The scarcity of specialized mental health services in these regions reflects this imbalance [37, 38].

The predominant state operation of PHC facilities, while instrumental in ensuring widespread basic healthcare coverage, often falls short in providing specialized care like AMH services. This is primarily due to limited resources and a focus on addressing more immediate and visibly pressing health concerns, such as infectious diseases or primary care needs. Mental health services, which require specialized training, infrastructure, and sustained investment, are often sidelined in resource allocation decisions.

Furthermore, the challenges of geographical accessibility in rural areas exacerbate the problem. Limited transportation options, long distances to PHC facilities, and the associated costs deter individuals from seeking care, particularly for conditions like mental health issues that may not be deemed as urgent as physical health ailments.

The state-driven healthcare models in Burkina Faso and Niger, despite their intent to provide broad coverage, might lack the flexibility and resources to adapt to the specific needs of different regions, particularly in terms of mental health. This calls for a reevaluation of healthcare strategies and policies, with an emphasis on equitable resource distribution that considers both geographic and specialized healthcare needs.

The challenges highlighted in Table 3 regarding the lack of essential skills and resources in West African health systems are critical in understanding the shortcomings in providing comprehensive AMH care. The absence of national guidelines in most PHC facilities is not just an operational oversight; it signifies a systemic neglect in recognizing and addressing AMH as a distinct and crucial aspect of public health.

The lack of standardized care protocols for AMH services leads to inconsistencies in treatment quality and effectiveness across different PHC facilities. Without national guidelines, healthcare providers may lack clear direction on best practices, leading to a potential disparity in the care adolescents receive. This inconsistency can undermine the trust and confidence in mental health services, further discouraging adolescents and their families from seeking necessary care.

The consistently low OPD attendance rates, particularly in Ghana and Burkina Faso, are indicative of broader issues beyond service availability. The stigma surrounding mental health conditions remains one of the most significant barriers. In many West African communities, mental health issues are often misunderstood, leading to social stigma and discrimination. This cultural barrier can prevent adolescents and their families from acknowledging mental health issues and seeking help [36].

Lack of awareness further compounds this issue. Many adolescents, and even adults, in these regions may not recognize the signs of mental health disorders or understand the importance of seeking treatment. Mental health education and awareness campaigns are crucial to change this narrative, ensuring that individuals are informed and empowered to seek help [39, 40].

Accessibility challenges also play a crucial role. In rural areas, where most of the population might reside, the physical distance to healthcare PHC facilities offering AMH services can be a significant hindrance. For many, the cost of transportation, coupled with the potential loss of daily wages, makes accessing mental healthcare services impractical or impossible.

### Policy recommendation

#### Enhancing accessibility of services

Efforts should be made to improve the physical and financial accessibility of mental health services. This could include introducing mobile health services, telemedicine, or subsidizing transport costs for rural populations. Financial barriers can be reduced through government subsidies or health insurance schemes that cover mental healthcare.

#### Development and implementation of national guidelines

Governments in these countries should prioritize the development and implementation of national guidelines for AMH services. These guidelines should standardize care across different regions and ensure that all adolescents have access to consistent and quality mental health services. This would also involve training healthcare providers in understanding and applying these guidelines effectively.

#### Increasing public awareness and reducing stigma

There is a need for widespread public health campaigns to increase awareness about mental health, particularly among adolescents. These campaigns should also aim to reduce the stigma associated with mental health issues by promoting understanding and acceptance. Collaborations with community leaders, schools, and religious institutions can be instrumental in changing public perceptions and attitudes towards mental health.

#### Expanding training of mental health professionals

Addressing the scarcity of trained mental health professionals requires both expanding training opportunities and creating incentives for professionals to work in underserved areas, particularly in rural regions. Specialized training programs in AMH should be established, and existing healthcare workers should be offered continuing education to improve their capacity to deal with mental health issues.

#### Integration of mental health into primary health care

Mental health services should be integrated into the broader primary healthcare system. This approach ensures that mental health is not treated in isolation but as part of comprehensive healthcare, making it easier for adolescents to access these services without stigma or discrimination.

## Conclusion

The study of AMH services in Burkina Faso, Ghana, and Niger has revealed significant gaps and disparities in the availability and quality of these crucial services. The findings indicate that less than 40% of primary healthcare centers across these countries offer AMH services, highlighting a substantial shortfall in meeting the mental health needs of this vulnerable population. This deficiency is further exacerbated by socio-cultural stigmas, economic barriers, and the scarcity of trained mental health professionals, which collectively impede access to and the effectiveness of these services.

Additionally, the study underscores the pronounced rural-urban divide in service provision, with rural areas being markedly underserved. The absence of national guidelines for AMH services and the low OPD attendance rates further emphasizes the systemic inadequacies and the need for urgent, targeted interventions.

In conclusion, this research calls for a comprehensive approach to reforming mental health services for adolescents in these countries. This approach should encompass policy reform, public awareness campaigns, enhanced training for healthcare professionals, and equitable resource distribution. Addressing these challenges is not only crucial for improving the mental health and well-being of adolescents in West Africa but is also fundamental to the socio-economic development of the region.

## Data Availability

The datasets used and/or analysed during the current study are available from the corresponding author on reasonable request.

## Abbreviations

AMH: Adolescent mental health
CHPS: Community-based health planning and services
OPD: Outpatient department
PHC: Primary health care
WHO: World Health Organization
PTSD: Post-traumatic stress disorder
AdoWA: Adolescent mental, sexual and reproductive health and wellbeing policy, program and primary care implementation priorities in West Africa
mhGAP: Mental health gap action programme
